# Delusional Infestation in Parkinson's Disease Secondary to Piribedil Escalation: An Uncommon Case Report

**DOI:** 10.7759/cureus.53631

**Published:** 2024-02-05

**Authors:** Aziz Ahizoune, Maha Ait Berri

**Affiliations:** 1 Department of Neurology, Military Hospital Moulay Ismail of Meknès, Sidi Mohamed Ben Abdellah University, Fez, MAR

**Keywords:** dopaminergic agonist, adverse event, piribedil, parkinson’s disease, delusional infestation

## Abstract

Delusional infestation (DI) is characterized by delusions of being infested by small microorganisms or even inanimate objects without any medical or microbiological evidence. The pathophysiology of DI is not well understood, and there are two types of DI: the primary form, where there is no underlying cause, and the secondary form, which is related to an associated psychiatric disorder, medical condition, or substance use. DI in Parkinson's disease (PD) is rarely reported, and most published cases are due to antiparkinsonian drugs. Piribedil is a dopaminergic agonist used for the symptomatic treatment of PD either as monotherapy or as adjuvant therapy with other antiparkinsonian treatments. We report the case of an 81-year-old man followed for PD at our institution who developed DI after piribedil dose escalation. When DI is secondary to an antiparkinsonian drug, the treatment of choice is based on withdrawing the implicated drug.

## Introduction

Delusional infestation (DI) is a psychotic disorder characterized by a delusional belief of being infested by small microorganisms (parasites, insects) or even inanimate objects without any medical or microbiological evidence of a true infestation. Several names have been used to describe this syndrome, like Ekbom's syndrome or delusions of parasitosis, but the most appropriate term, according to experts, is DI [1]. The patients report abnormal skin feelings, such as tingling, itching, or biting sensations that could provoke self-inflicted skin lesions [2].

The neurobiological mechanisms of this disorder are not fully understood, and there are two types of DI: primary and secondary forms. When the delusions are caused by either an underlying medical condition, medication, or illicit drugs, the condition is termed secondary DI. In contrast, when there are no underlying causes, DI is referred to as the primary form [1,3].

The most commonly reported drugs associated with DI are amantadine, dopaminergic agonists, amphetamine, pemoline, methylphenidate, and cocaine [1,2]. DI is rarely reported in Parkinson's disease (PD) patients, with only a few cases published in the literature, mainly following the use of antiparkinsonian drugs. Piribedil is a dopaminergic agonist used for the symptomatic treatment of PD and is rarely associated with DI. Here, we report a case of DI following an escalation in the dose of piribedil in a patient with PD.

## Case presentation

An 81-year-old male patient with no pathological history, seen in the neurology department since 2014 for Parkinson's disease, presented with rest tremor of the left hand, extrapyramidal hypertonia with bradykinesia. The patient had a slowly progressive course of the disease without psychiatric symptoms or memory problems. He was on benserazide/levodopa 25/100 mg four times/day and piribedil 50 mg/day for a long time.

**Figure 1:**
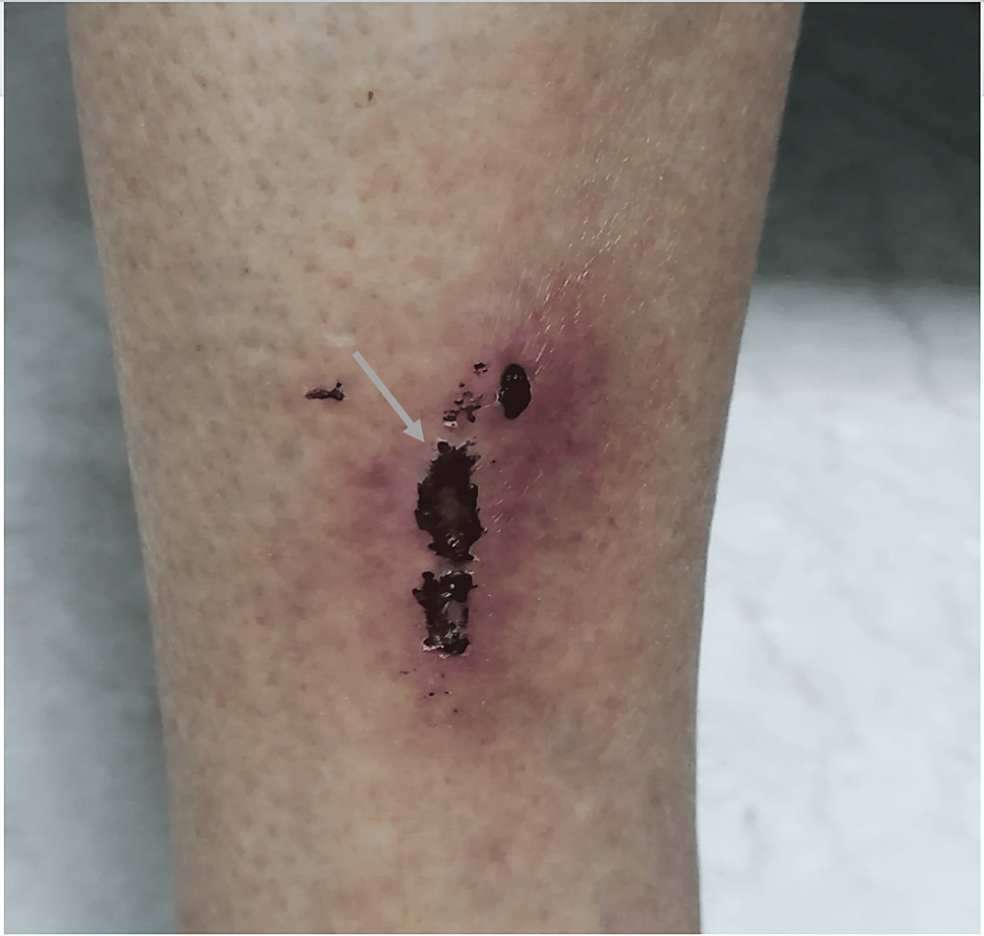
Excoriation lesions on the anterior part of the right leg with repeated manipulations leading to an increase in the size of the lesions

One month ago, he presented an increase in the intensity of the rest tremor, which necessitated an increase in the dose of piribedil from 50 mg/day to 150 mg/day without modifying the dose of benserazide/levodopa. Two weeks after piribedil escalation, he began to report that his skin, especially on his limbs, head, and trunk, was infested with crawling worms that were moving on his skin and causing pruritus sensations. As a result, he often tried to remove the parasites by scratching his skin, causing skin lesions, particularly scars and excoriations lesions (Figure 1).

**Figure 2:**
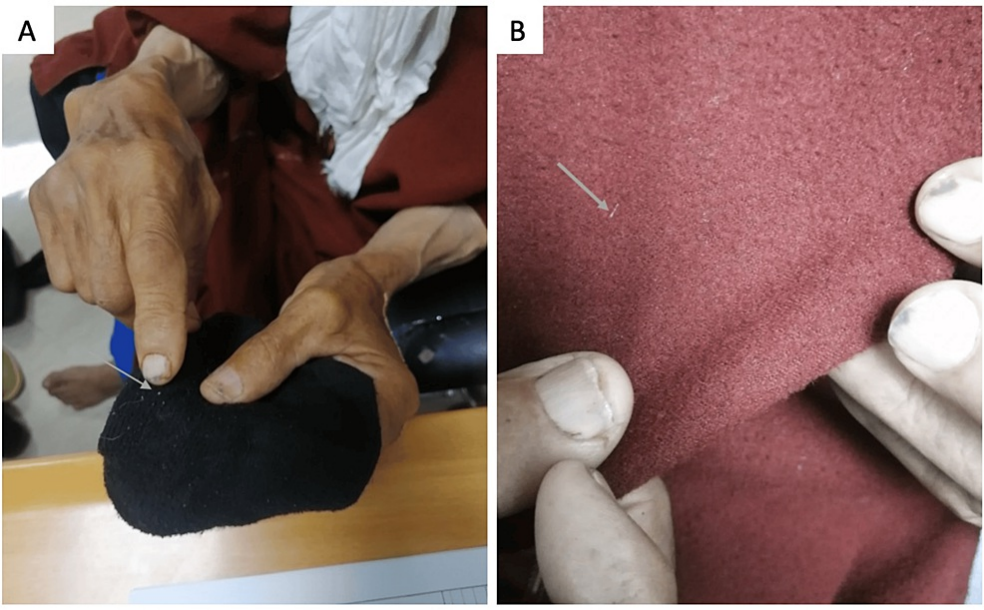
The patient tried to convince us that these small white fibers are the etiology of the infestation A: small whitish filament on his socks; B: white filament on his clothes (djellaba)

He had collected these worms in a box but hadn't brought them with him, and during the assessment, he tried to convince us that the small white fibers on his clothes were the cause of the infestation (Figure 2). He didn't complain of any other psychotic symptoms or neurological deterioration. Neurological examination revealed a Parkinsonian syndrome predominantly on the left side, with no disorientation or cognitive impairment. A dermatological assessment performed by a dermatologist revealed the presence of excoriated skin lesions with no signs of infestation or infection. MRI of the brain revealed no significant abnormalities. Complete blood count, C-reactive protein, serum creatinine, and glycemia were normal.

The main differential diagnoses were psychiatric disorders such as schizophrenia, obsessive-compulsive disorder, and major depressive disorder with psychotic symptoms, which were excluded due to the advanced age of the patient, the absence of anxiety-depressive manifestations, and other psychotic symptoms. The diagnosis of delusional infestation was evident in our situation based on the clinical manifestations. The increased dose of piribedil was the most likely cause of the DI. For this reason, we decided to stop piribedil, but the other medication remained unchanged. The delusions gradually disappeared two weeks later, confirming our clinical diagnosis. After the DI was resolved, we increased the dose of benserazide/levodopa to control the symptoms of PD. The diagnosis of a psychotic disorder, in the form of DI, triggered by an increase in the dose of piribedil was retained in this context.

## Discussion

Piribedil is a dopaminergic agonist that has been used worldwide for the treatment of Parkinson's disease since 1970. It may be used as monotherapy or as adjuvant treatment with other antiparkinsonian medications [4]. Several side effects have been reported with piribedil, including mainly gastrointestinal disturbances, orthostatic hypotension, sleep attacks, somnolence, and neuropsychiatric features dominated by visual hallucinations [5].

The most common psychotic manifestations in PD are visual hallucinations, delusions, and illusions, which occur in 20-30% of cases [6]. These symptoms are seen mainly in the advanced stages of the disease and are often secondary to antiparkinsonian drugs and the underlying pathological process of PD [7]. Our patient had been taking piribedil for a long time without any psychotic events. However, the increase in piribedil dose and the onset of DI suggest a predisposition associated with advanced PD and exceeding the threshold of tolerance to piribedil.

The pathophysiology of DI is not fully clarified, and experts suggest that interactions between dopaminergic, cholinergic, and serotonergic systems underlie the emergence of psychotic manifestations in PD patients [7]. In addition, some studies have pointed out that reduced dopamine transport within the striatum, with an increase in its extracellular levels, may lead to the development of delusions [8]. Structural lesions, mainly involving the striatum, have been implicated as a secondary organic cause of DI, and that's why our patient underwent a brain MRI to rule out a structural lesion [1,8].

The description of DI in patients with PD is uncommon, and there are only a few cases in the literature, principally as a result of antiparkinsonian drugs. The most common medicines involved in these patients are amantadine, anticholinergics, monoamine oxidase B inhibitors, dopamine agonists, catechol-o-methyl-transferase (COMT) inhibitors, and L-DOPA [9]. In general, the main complaints of these patients are infestations by bugs, spiders, webs, and whitish fibers [9,10]. In our context, the patient believed he was infested by whitish fibers, which caused pruritus. Typically, patients may use their fingers, nails, or tools to relieve the itching. This scratching can lead to skin damage, such as ulcerations, as in our case, and sometimes to lichenification and severe mutilations [1].

However, regarding the involvement of piribedil in the onset of DI, as seen in our case, Kölle et al. reported a unique case of DI in a 61-year-old Caucasian woman who had been treated for PD for five years with L-DOPA/decarboxylase, ropinirole and pramipexole [10]. Following a worsening of her condition, piribedil 200 mg daily was introduced instead of pramipexole. On the fourth day, she developed DI, which was secondary to the piribedil add-on. Whereas, in our case the patient tolerated a dose of 50 mg per day for a long time but did not tolerate a dose of 150 mg per day. In Kölle et al. case, DI disappeared 12 days after stopping piribedil [10], which is similar to what we observed in our case.

Most reported cases of DI following PD treatment had a resolution of symptoms after withdrawal of the drug concerned, as shown in our observation [9,11]. Antipsychotic treatments are used for the symptomatic management of patients with primary DI [1]. However, in drug-induced forms, treatment is essentially based on stopping the drug involved [9].

## Conclusions

Parkinson's disease is a frequent neurodegenerative disease in the elderly, and the use of dopaminergic agonists is part of the therapeutic management of these patients. Delusional infestation is a psychotic disorder that impairs patients' quality of life, and is often secondary to antiparkinsonian treatment in the setting of PD. Cases of DI following the use of piribedil are rare, but clinicians should be aware of the possibility of DI in patients with PD. The management of this psychotic disorder when secondary to antiparkinsonian drugs is to discontinue the causal drug.
